# Scopolin ameliorates high-fat diet induced hepatic steatosis in mice: potential involvement of SIRT1-mediated signaling cascades in the liver

**DOI:** 10.1038/s41598-017-02416-6

**Published:** 2017-05-22

**Authors:** Ahyoung Yoo, Vikram P. Narayan, Eun Young Hong, Wan Kyunn Whang, Taesun Park

**Affiliations:** 1 Department of Food and Nutrition, Brain Korea 21 PLUS Project, Yonsei University, 50 Yonsei-ro, Seodaemun-gu, Seoul, 03722 South Korea; 2 Pharmaceutical Botany Laboratory, College of Pharmacy, Chung-Ang University, Heukseok-dong, Dongjak-gu, Seoul, 06974 South Korea

## Abstract

The present study aimed to investigate whether scopolin exhibits beneficial effects on high-fat diet (HFD)-induced hepatic steatosis in mice. The involvement of sirtuin 1 (SIRT1) as a molecular target for scopolin was also explored. Scopolin decreased the Km of SIRT1 for p53 and nicotinamide adenine dinucleotide without altering Vmax in a cell-free system. Scopolin alleviated oleic acid-induced lipid accumulation and downregulation of SIRT1 activity in HepG2 cells, and these beneficial effects of scopolin were abolished in the presence of SIRT1 inhibitor. Mice administered 0.02% scopolin for 8 weeks exhibited improved phenotypes of HFD-induced hepatic steatosis along with increased hepatic SIRT1 activity and protein expression. Scopolin resulted in increased deacetylation of sterol regulatory element-binding protein 1c with subsequent downregulation of lipogenic genes, and enhanced deacetylation of protein peroxisome proliferator-activated receptor-γ coactivator 1α with upregulation of fatty acid oxidation genes in livers. Scopolin also enhanced deacetylation of nuclear factor-kappa enhancer binding protein and liver kinase B1 (LKB1), facilitating LKB1/AMP-activated protein kinase signaling cascades. Scopolin attenuated hepatic steatosis through activation of SIRT1-mediated signaling cascades, a potent regulator of lipid homeostasis. Increased hepatic SIRT1 activity and protein expression appeared to be associated with these beneficial effects of scopolin.

## Introduction

Hepatic steatosis, defined as the accumulation of triglycerides (TGs) in hepatocytes, results from an imbalance between pathways leading to TG synthesis and catabolism. In hepatocytes with prolonged TG accretion, nuclear factor-kappa enhancer binding protein (NF-κB) that functions as a proinflammatory master switch is activated, leading to a subacute inflammatory response. These pathways involved in the regulation of hepatic lipid metabolism and inflammation share a common regulator, sirtuin 1 (SIRT1) that is a nicotinamide adenine dinucleotide (NAD^+^)-dependent protein deacetylase localized exclusively in the nucleus^1^. SIRT1 has been shown to deacetylate many non-histone proteins in hepatocytes, including sterol regulatory element-binding protein 1c (SREBP1c)^2–4^, peroxisome proliferator-activated receptor-γ coactivator 1α (PGC-1α)^5^, liver kinase B1 (LKB1)^6^, and NF-κB^7, 8^. Deacetylated SREBP1c transcription factor results in the reduced expression of lipogenic genes such as acetyl-CoA carboxylases (ACC) and fatty acid synthase (FAS)^9, 10^, whereas deacetylation of PGC-1α enhances its function upregulating the transcription of carnitine palmitoyltransferase 1 (CPT1) needed for mitochondrial fatty acid oxidation^11–13^. Once LKB1 is deacetylated, it is translocated to the cytoplasm and then engaged in the phosphorylation of AMP-activated protein kinase (AMPK) which leads to both decreased lipogenesis *via* the mammalian target of rapamycin (mTOR)/ liver X receptor α (LXRα) signaling cascade and increased fatty acid oxidation *via* ACC phosphorylation^14^.

**Figure 1:**
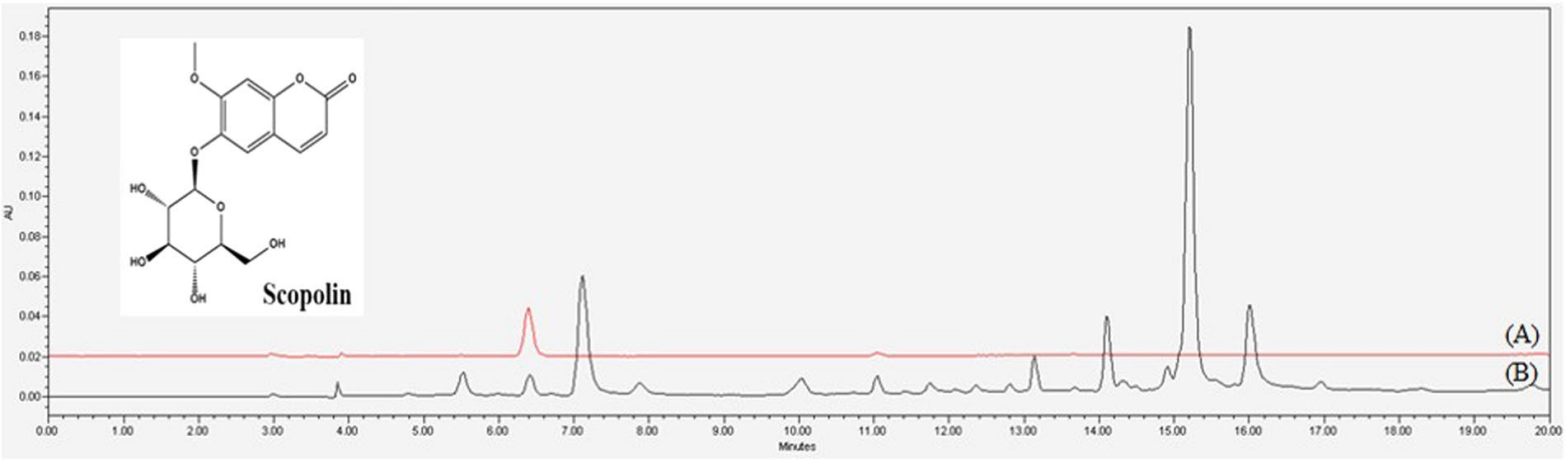
HPLC chromatogram and chemical structure of scopolin. (**A**) HPLC chromatogram of the isolated compound scopolin and its structure. (**B**) HPLC chromatogram of *Artemisia iwayomogi* extracts.

Scopolin (C_16_H_18_O_9_, Fig. 1) is a coumarin compound found in a wide variety of plant species including *Artemisia iwayomogi, Erycibe obtusifolia, Santolina oblongifolia*, and *Scopolia carniolica* ^15–18^. Scopolin isolated from *S. oblongifolia* has been shown to inhibit the release of eicosanoids from ionophore-stimulated mouse peritoneal macrophages^19^. Scopolin extracted from *E. obtusifolia* stems ameliorated adjuvant-induced arthritis in rats by down-regulating proinflammatory and proangiogenic cytokines, such as vascular endothelial growth factor, basic fibroblast growth factor-2, and interleukin-6 (IL-6)^20^. Furthermore, Rollinger *et al*.^18^ showed that scopolin isolated from *S. carniolica* Jaqc increased the extracellular acetylcholine concentration, which is responsible for regulating learning and memory, in rat brains up to 300% compared to the basal release, potentially reducing the risk of Alzheimer’s disease.

Dietary intervention with several bioactive phytochemicals has been proven to be an effective approach to reducing the risk of hepatic steatosis^21–24^. We previously reported that *A. iwayomogi* extract, mainly consisting of scopolin (1.21% w/w) along with other compounds such as scopoletin (0.38% w/w) and acetophenone glycoside (0.26% w/w) reversed high-fat diet (HFD)-induced abnormal increments in visceral adiposity, hepatic steatosis, and hyperlipidemia in mice^15^. A more recent study has demonstrated that scopoletin, a molecule lacking the glucopyranoside chain of scopolin, exhibited protective effects against ethanol-induced hepatic lipid accumulation in HFD-fed mice^25^. Nevertheless, the protective role of scopolin against adiposity and metabolic diseases has not yet been reported in *in vitro* or in *vivo* systems. The present study investigated whether scopolin isolated from *A. iwayomogi* exerts beneficial effects on HFD-induced hepatic steatosis in mice. We explored the potential involvement of SIRT1 as a molecular target for scopolin in the process of reducing hepatic lipid accumulation.

## Results

### Nuclear magnetic resonance (NMR) structure elucidation and HPLC analysis of scopolin

Isolated white amorphous scopolin powder was measured by ^1^H-NMR (600 MHz) and ^13^C-NMR (150 MHz) on a Varian Gemini 2000 spectrometer (Varian Inc., Palo Alto, CA, USA). ^1^H-NMR (DMSO-*d*_6_), δ 7.97 (1H, d, *J* = 9.6 Hz, H-4), 7.29 (1H, s, H-5), 7.15 (1H, s, H-8), 5.09 (1H, d, *J* = 9.0 Hz, H-3), 5.06 (1H, d, *J* = 7.2 Hz, H-1′), 3.79 (3H, s, OCH_3_), 3.47 (1H, m, H-2′), 3.43 (1H, dd, *J* = 9, 12 Hz, H-5′), 3.33 (2H, m, H-3′, H-4′), 3.18 (1H, m, H-6′b), 2.52 (1H, m, H-6′a); ^13^C-NMR (DMSO-*d*_6_), δ 162.2 (C-2), 151.4 (C-7), 150.5 (C-9), 147.6 (C-6), 145.9 (C-4), 114.9 (C-3), 113.9 (C-10), 111.3 (C-5), 101.1 (C-1′), 78.6 (C-3′), 78.1 (C-5′), 74.5 (C-2′), 71.1 (C-4′), 62.1 (C-6′), 57.7 (OCH_3_). The structure of scopolin (C_16_H_18_O_9_) is shown in Fig. 1.

The purity of this compound was assessed using HPLC analysis, which showed a single peak at a retention time of 6.3 min (Fig. 1A). The purity of this peak was determined to be 99.8%. The HPLC chromatogram of the *A. iwayomogi* ethanol extracts revealed that the scopolin yield was 0.97 ± 0.12% (w/w) (Fig. 1B).

### Scopolin exerts its anti-lipogenic activity via SIRT1 in HepG2 cells

**Figure 2:**
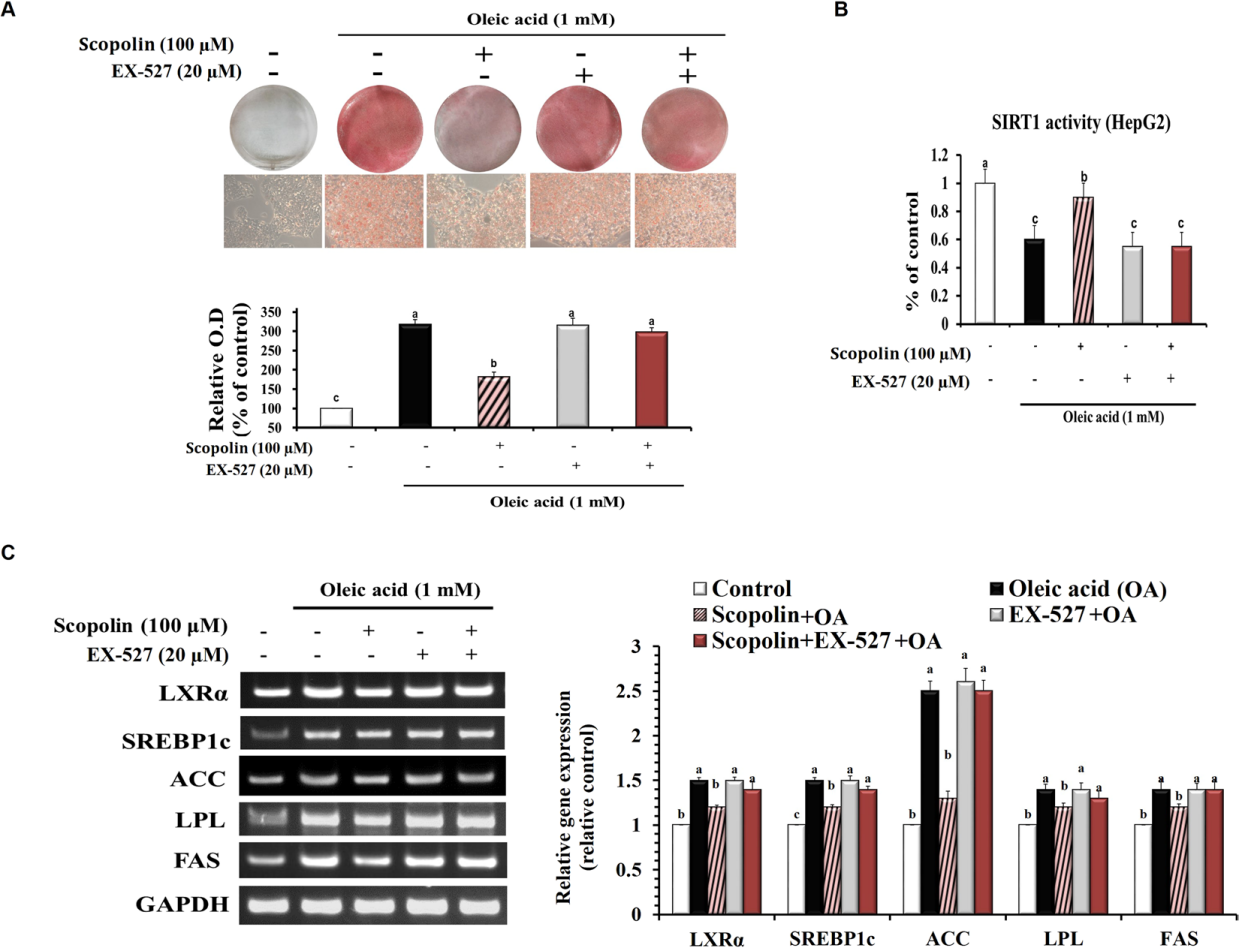
Scopolin exerts its anti-lipogenic activity via SIRT1 in HepG2 cells. Cells were exposed to oleic acid (1 mM), scopolin (100 μM), and SIRT1-specific inhibitor EX-527 (20 μM) for 24 hr. (**A**) Oil-Red O staining of cells with different treatments. Images are representative of at least three independent experiments. Original magnification 400x. (**B**) Catalytic activity of SIRT1 in HepG2 cells. (**C**) The expression of lipogenic-related genes was determined by RT-PCR and normalized to that of GAPDH. The full-length gels are presented in Supplementary Fig 1. Data means from three independent experiments. Different letters indicate statistical significance, p < 0.05.

Scopolin (100 μM) effectively reversed oleic acid-induced lipid accumulation in HepG2 cells, as shown by reduced Oil-Red O stained lipid accumulation, and this beneficial effect of scopolin was blocked in the presence of the SIRT1 inhibitor EX-527 (Fig. 2A). Next, the effect of scopolin on SIRT1 activity was determined in HepG2 cells treated with oleic acid in the presence or absence of EX-527. Scopolin effectively restored the SIRT1 deacetylase activity downregulated by oleic acid in HepG2 cells, and this effect was completely abolished in the presence of EX-527 in the culture medium (Fig. 2B). Furthermore, significant upregulation of lipogenic transcription factors, such as LXRα, SREBP1c, and their target genes, such as ACC, lipoprotein lipase (LPL), and FAS, was observed in cells treated with oleic acid. Scopolin significantly reversed these oleic acid-induced upregulations of lipogenic genes, again, these beneficial effects were not observed when the cells were treated with EX-527 (Fig. 2C).

### Scopolin alleviates hepatic steatosis in HFD-fed mice

**Figure 3:**
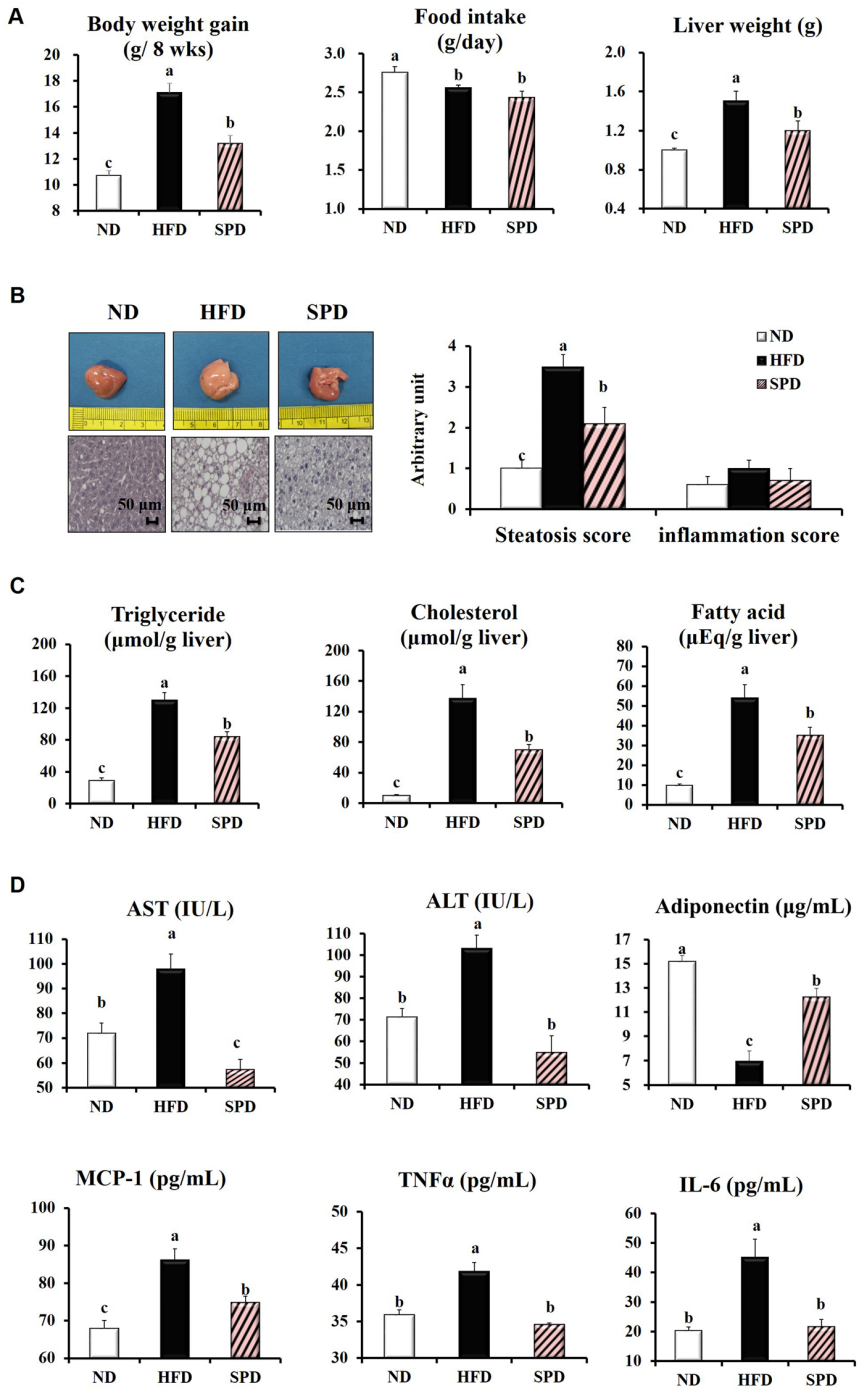
Scopolin alleviates hepatic steatosis in HFD-fed mice. C57BL/6 N mice were fed a ND, HFD, and SPD for 8 weeks. (**A**) Changes in body weight, food intake, and liver weight. (**B**) Representative pictures of livers and H&E staining for liver (scale bar = 50 μm), and hepatic steatosis and inflammation scores. (**C**) Hepatic TG, cholesterol, and fatty acid concentrations. (**D**) Plasma AST and ALT activities and plasma adiponectin, MCP-1, TNFα, and IL-6 levels. Data shown are means ± SEM (n = 8). Bars with different letters differ significantly (p < 0.05).

Scopolin administration for 8 weeks significantly decreased the body weight gain in mice fed the HFD (−23%, *p* < 0.05) without affecting food intake (Fig. 3A). SPD-fed mice also displayed lower gross liver weight compared with HFD-fed mice (−14%, *p* < 0.05, Fig. 3A). There were paramount differences in color and size of whole livers among ND-, HFD-, and SPD-fed mice. Our photographic images and H&E staining data showed that the remarkable lipid droplets appeared in the livers of HFD-fed mice were effectively diminished on scopolin administration. To confirm this finding, steatosis and inflammation scores were evaluated in all H&E staining images of liver tissues from ND-, HFD-, and SPD-fed mice. The HFD-induced elevation of the hepatic steatosis score was significantly normalized in the livers of SPD-fed mice. Although the hepatic inflammation scores followed a pattern similar to that of the steatosis scores, there were no statistically significant difference among the groups (Fig. 3B).

HFD feeding for 8 weeks successfully induced fatty liver and liver injury in mice, which were manifested by significant increase in hepatic TG, cholesterol, and fatty acid concentrations and plasma AST, and ALT activities compared with those of ND-fed mice (Fig. 3C and D). In the meantime, scopolin significantly reversed the HFD-induced hepatic accumulation of TG, cholesterol, and fatty acid by 35%, 49%, and 35%, respectively (Fig. 3C), as well as lowering the plasma AST and ALT activities by 41% and 47%, respectively (Fig. 3C and D). Furthermore, scopolin significantly reversed the HFD-induced decrease in plasma adiponectin levels by 76%. HFD-induced increases in plasma MCP-1, TNFα, and IL-6 levels were significantly reduced by scopolin administration (Fig. 3D).

### Scopolin activates SIRT1 by decreasing its Michaelis-Menten constant (Km)

**Figure 4:**
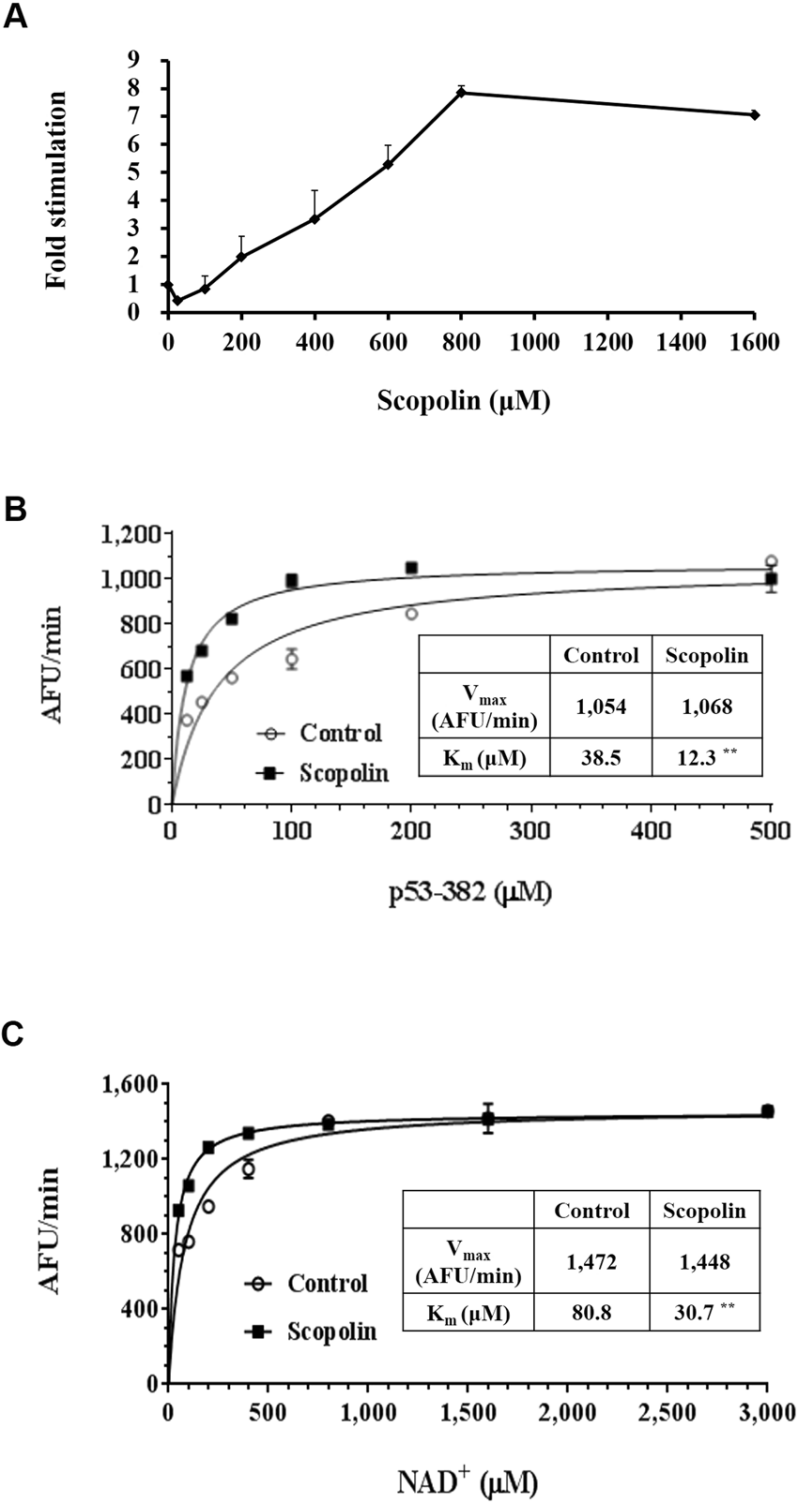
Scopolin activates SIRT1 by decreasing its Km. (**A**) Scopolin dose-response curve of the SIRT1 catalytic rate. (**B**) SIRT1 initial rate with 3 mM NAD^+^, as a function of the p53-382 acetylated peptide concentration in the presence (open circles) or absence (closed squares) of 800 μM scopolin. Lines represent nonlinear least-squares fits to the Michaelis-Menten equation. Km (control, open circles) = 38.5 μM, Km (plus scopolin, closed squares) = 12.3 μM; Vmax (control, open circles) = 1,054 AFU/min, Vmax (plus scopolin, closed squares) = 1,068 AFU/min. (**C**) SIRT1 initial rate at 500 μM p53-382 acetylated peptide, as a function of the NAD^+^ concentration, in the presence (open circles) or absence (closed squares) of 800 μM scopolin as in (**B**). Km (control, open circles) = 80.8 μM, Km (plus scopolin, closed squares) = 30.7 μM; Vmax (control, open circles) = 1,472 AFU/min, Vmax (plus scopolin, closed squares) = 1,448 AFU/min. Data represent means from three independent experiments. Different letters indicate statistical significance, p < 0.05.

To evaluate SIRT1 as a molecular target for scopolin, deacetylase activity by human SIRT1 was measured over a range of scopolin concentrations with fixed levels of both acetylated peptide substrate and NAD^+^ in a cell-free system. Dose–response experiments showed that scopolin doubled the rate of deacetylation by SIRT1 at approximately 200 μM; SIRT1 activity was saturated at 600–800 μM scopolin, exhibiting an 8-fold stimulation (Fig. 4A). Next, the rate of deacetylation by SIRT1 was measured with various concentrations of a p53 peptide substrate or the coenzyme NAD^+^ in the presence or absence of 800 μM scopolin. The velocity of enzyme-catalyzed reaction at infinite concentration (Vmax) of either acetylated peptide substrate or NAD^+^ was not affected by scopolin. Whereas, scopolin significantly lowered the Km of both acetylated peptide substrate (62% reduction) and NAD^+^ (68% reduction) (Fig. 4B and C). These results suggest that scopolin may work as an activator of SIRT1 by reducing the affinity of this enzyme for both acetylated peptide substrate and NAD^+^.

### Scopolin activates SIRT1-mediated signaling cascades in the liver of mice

**Figure 5:**
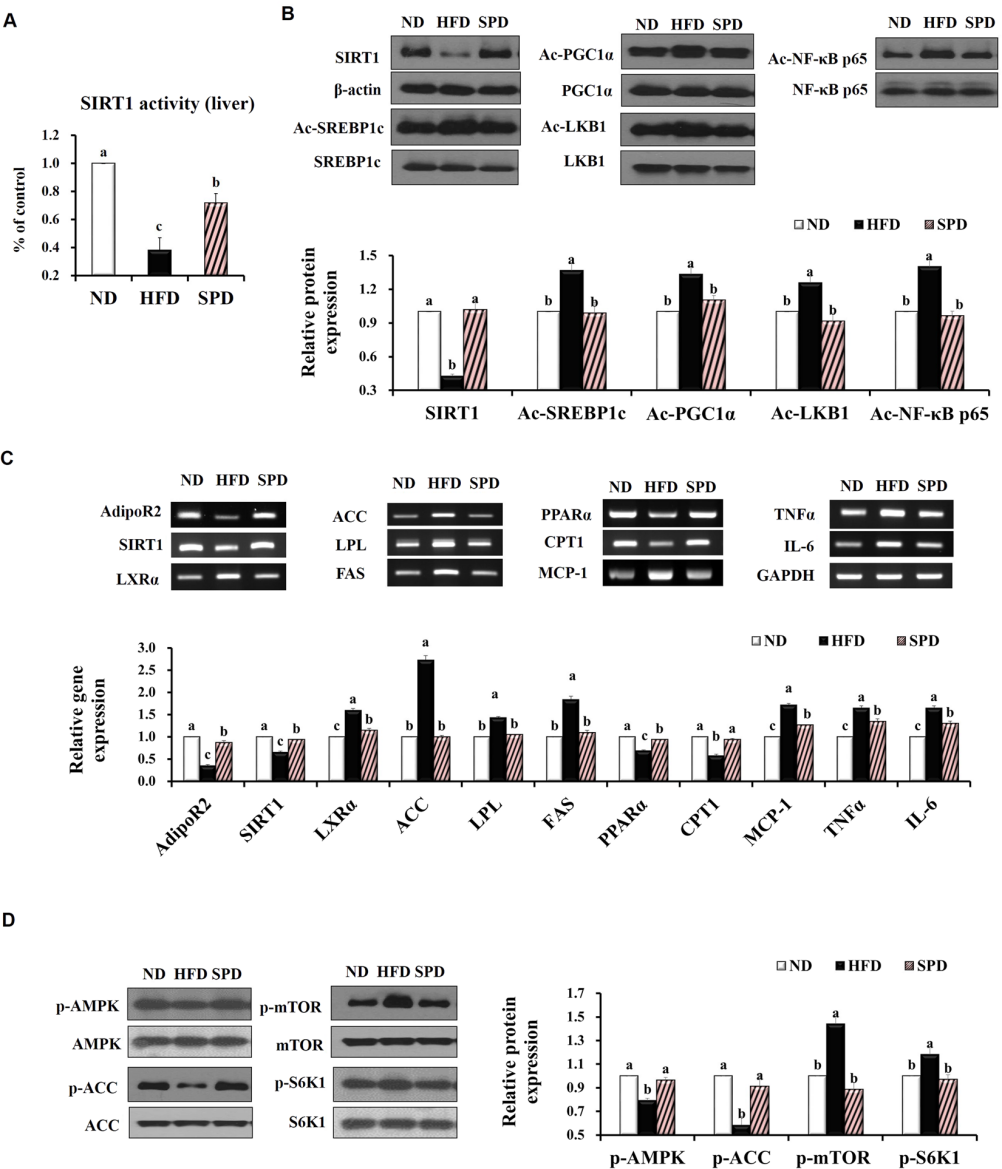
Scopolin activates SIRT1-mediated signaling cascades in mouse livers. (**A**) Catalytic activity of SIRT1 in the liver of mice fed experimental diets. (**B**) Representative Western blots bands and quantitative comparison of SIRT1 protein levels (normalized by corresponding β-actin expression level) and acetylated SREBP1c, PGC-1α, LKB1, and NF-κB levels (normalized by corresponding total SREBP1c, PGC-1α, LKB1, and NF-κB expression levels). (**C**) Representative RT-PCR product bands and quantitative comparison of mRNA expression of AdipoR2, SIRT1, LXRα, ACC, LPL, FAS, PPARα, CTP1, MCP-1, TNFα, and IL-6 in livers from different groups. GAPDH was used as a normalization control. (**D**) Representative Western blots bands and quantitative comparison of p-AMPK, p-ACC, p-mTOR, and p-S6K1 levels in livers from different groups, normalized by corresponding total AMPK, mTOR, S6K1, and ACC expression levels. The full-length blots/gels are presented in Supplementary Figs 2–4. Results are means from an n = 8 ± SEM of three independent experiments (*n* = 2 or 3 per experiment) for each group. Different letters indicate statistical significance, *p* < 0.05.

SIRT1 deacetylase activity was measured in the nuclear extracts of liver tissues from animals fed experimental diets. HFD feeding for 8 weeks significantly decreased hepatic SIRT1 activity compared with that in ND-fed mice, while scopolin significantly restored HFD-induced downregulation of SIRT1 activity (Fig. 5A). Immunoblot results confirmed that scopolin administration significantly increased SIRT1 hepatic protein levels. In conjunction with scopolin-induced activation and abundance of hepatic SIRT1 enzyme, acetylated protein levels of SREBP1c, PGC-1α, LKB1, and NF-κB were all downregulated in the livers of SPD-fed mice compared with HFD-fed mice (Fig. 5B).

Scopolin administration significantly reversed the HFD-induced downregulation of hepatic adiponectin receptor 2 (AdipoR2) and SIRT1 genes. mRNA expression of lipogenic genes, such as LXRα, ACC, LPL, and FAS, was significantly downregulated, whereas the expression of peroxisome proliferator-activated receptor alpha (PPARα) and CPT1, regulators of fatty acid oxidation, was significantly upregulated in the livers of SPD mice than in the HFD mice. Hepatic mRNA expression of the proinflammatory cytokines, MCP-1, TNFα, and IL-6 was all significantly downregulated by scopolin administration (Fig. 5C). Western blot analysis showed increased p-AMPK and p-ACC expression levels in the livers of SPD-fed mice, along with decreased p-mTOR and p-S6K1 levels, compared with HFD-fed mice (Fig. 5D).

## Discussion

In this study, the mice were fed the 0.02% scopolin-supplemented diet (equivalent to 20 mg/kg body weight) which are corresponding to an intake of approximately 1.6 mg/kg body weight (97 mg/60-kg person), when calculated on the basis of normalization to body surface area as recommended by Reagan-Shaw *et al*.^26^ and the US Food and Drug Administration (http://www.fda.gov/cder/cancer/animalframe.htm). On the basis of our findings that the yield of scopolin isolated from dried *A. iwayomogi* extract was 0.97% in the present study, a daily human dose of 97 mg scopolin would correspond to approximately 9.9 g extract of *A. iwayomogi*. The toxicity of scopolin has yet to be reported. In rats, the no observed adverse effect level (NOAEL) value of orally administered *A. iwayomogi* Kitamura extract was reported as exceeding 2,000 mg/kg/day^27^, which is equivalent to 19.5 g/60-kg human/day. The absorption, pharmacokinetics, bioavailability, biodistribution, and metabolism of scopolin are unknown. A scopolin pharmacokinetics study in rats revealed that after a single oral administration of scopolin (100 mg/kg), the mean maximum plasma concentration of scopolin was 3.33 μM/ mL, the mean Tmax was 20 min, and the mean elimination half-life was 33 min^16^.

The present study found that scopolin significantly alleviated hepatic steatosis induced by HFD in mice, as demonstrated by the improved parameters of lipid droplets in liver tissues, hepatic lipid levels, and plasma AST and ALT activities, and adiponectin levels as well. These beneficial effects of scopolin on hepatic steatosis appear to be associated with increased deacetylation of the SREBP1c protein with subsequent downregulation of lipogenic genes (LXRα, ACC, LPL, and FAS), along with increased deacetylation of the PGC-1α protein with upregulation of genes involved in fatty acid oxidation (PPARα, and CPT1). Furthermore, scopolin increased the deacetylation of a proinflammatory transcription factor, NF-κB, with subsequent downregulation of its target genes (MCP-1, TNFα, and IL-6), in the absence of inflammatory phenotypes. HFD-induced fatty liver diseases can progress from simple steatosis to nonalcoholic steatohepatitis (NASH, fatty changes with inflammation and hepatocellular injury or fibrosis). It has repeatedly been observed by us, and by other investigators that mice fed the HFD for 10 weeks showed simple steatosis with the absence of necrosis or signs of inflammation. Moreover, HFD feeding for 10 weeks significantly elevated levels of serum ALT and AST, as well as the expression of hepatic pro-inflammatory cytokines such as TNF-α and MCP-1^28-30^. The latter is considered as an early event associating hepatic lipogenesis and inflammatory stress. Although NASH did not develop in our 8-week HFD-feeding protocol, the hepatic expression of pro-inflammatory cytokines could have facilitated the deterioration of steatosis to NASH if the experiment had been conducted for a longer duration.

The attenuation in hepatic steatosis in mice treated with scopolin could be associated with their decreased visceral adiposity and circulating lipid profile as we previously showed that *A. iwayomogi* extract which is majorly composed of scopolin, reduced body weight gain and hepatic lipid levels in mice fed a high-fat diet^15^. At the same time, our *in vitro* observation that scopolin (100 µM) effectively reversed oleic acid-induced lipid accumulation in HepG2 cells raised the possibility that scopolin could reduce hepatic lipids independent of alterations in visceral adiposity (Fig. 2A). On the basis of the finding that the hepatic lipid-lowering effect of scopolin in mice fed a HFD is accompanied by simultaneous regulation of lipogenesis, fatty acid oxidation, and inflammatory conditions, we may speculate the possibility that SIRT1 might be one of the molecular targets of scopolin.

SIRT1 is one of seven mammalian homologs of Sir2 that catalyzes NAD^+^-dependent protein deacetylation, yielding nicotinamide and *O*-acetyl-ADP-ribose along with the deacetylated lysine^31^. Several studies in a number of transgenic mouse models have revealed essential roles for this metabolic sensor in response to changes in cellular energy states^32^. For example, HFD-fed mice with a heterozygous deletion of SIRT1 (SIRT1^+/−^) develop obesity and insulin resistance^33^ and exhibit increased lipid accumulation and inflammation in the liver and adipose tissues^34^. Complementary to these findings, transgenic mice with a 2–4-fold overexpression of SIRT1 show lower lipid-induced inflammation, better glucose tolerance, and protection from hepatic steatosis^8^. Recently, SIRT1 has also been shown to be a potential therapeutic target for treatment of fatty liver disease^35, 36^. Liver-specific deletion of SIRT1 in mice markedly promotes the development of hepatic steatosis, independent of the presence of obesity, insulin resistance and inflammation^5, 37^.

Notably, direct or indirect evidences obtained from a cell-free system, HepG2 cells, and a hepatic steatosis the mouse model consistently supports the notion that scopolin can act as an activator of SIRT1 deacetylation. To address the mechanism of scopolin activating SIRT1 enzyme, we determined the effect of scopolin on the Vmax and Km of human SIRT1 for its acetylated peptide substrate p53 and cosubstrate NAD^+^ in a cell-free system. Scopolin decreased the Km of SIRT1 for p53 and NAD^+^ with no effect on the Vmax (Fig. 4). As scopolin acts only on Km, it can be classified as an allosteric effector, which may indicate that only the substrate-binding affinity of the enzyme has been altered. In HepG2 cells, oleic acid-induced lipid accumulation, downregulation of SIRT1 activity, and expression of lipogenic genes were reversed by scopolin; these positive effects of scopolin were abolished in the presence of a SIRT1 inhibitor, indicating that scopolin exerts its anti-lipogenic effects through SIRT1 (Fig. 2). Furthermore, these *in vitro* results correspond to our *in vivo* findings that hepatic SIRT1 enzyme activity was increased by scopolin administration in HFD-fed mice (Fig. 5A).

**Figure 6:**
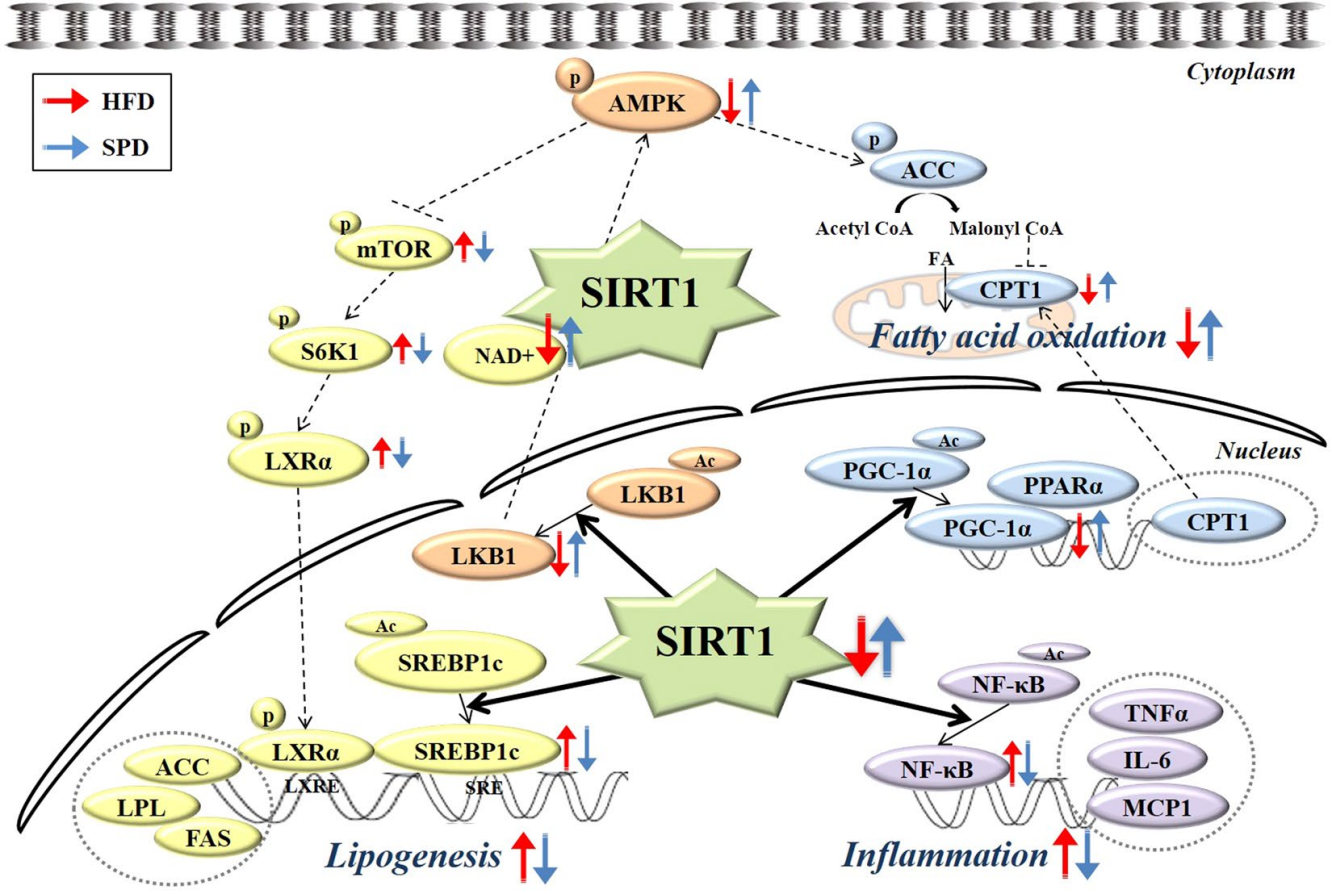
Proposed mechanism for the protective effects of scopolin against hepatic steatosis in mice: Key lipid regulatory pathways influenced by SIRT1 deacetylase activity. The crosstalk between various signaling pathways allows SIRT1 to function as a potent regulator of lipid homeostasis in liver tissues. As depicted, SIRT1 activation in response to scopolin can lead to increased deacetylation of SREBP1c protein in the liver with subsequent downregulation of lipogenic genes (LXRα, ACC, LPL, and FAS) along with increased deacetylation of PGC-1α protein with upregulation of genes involved in fatty acid oxidation (PPARα and CPT1). Alternatively or synergistically, scopolin-induced SIRT1 activation can lead to the deacetylation of LKB1 protein in the liver with consequent upregulation of p-AMPK and p-ACC, and downregulation of p-mTOR and p-S6K1, which would lead to increased fatty acid oxidation and decreased lipogenesis, respectively. SIRT1 activation can also lead to the deacetylation of NF-κB, a proinflammatory master switch with subsequent downregulation of MCP-1, TNFα, and IL-6.

Growing evidence suggests an association between SIRT1 and AMPK signaling^6, 38–40^. Activated SIRT1 acts upstream of AMPK signaling by modulating LKB1, an upstream AMPK kinase, suggesting that stimulation of SIRT1/LKB1/AMPK signaling may serve as a key mechanism for lipid metabolism in hepatic cells^6^. Indeed, in the present study, scopolin increased the deacetylation of LKB1 protein and resulted in subsequent upregulation of p-AMPK and p-ACC expression and downregulation of p-mTOR and p-S6K1, which would lead to increased fatty acid oxidation and decreased lipogenesis, respectively (Fig. 6). Therefore, considering the regulatory function of the SIRT1/LKB1/AMPK signaling pathway in hepatic lipid metabolism, we cannot conclude unequivocally that SIRT1-induced deacetylation of SREBP1c and PGC-1α is solely responsible for scopolin’s effects attenuating lipogenesis and enhancing fatty acid oxidation as illustrated in Fig. 6.

It is not entirely clear whether the protective effect of scopolin against HFD-induced hepatic steatosis is mediated primarily through the allosteric activation of SIRT1 in *vivo*. It is intriguing that besides the SIRT1 activity, the abundance of hepatic SIRT1 protein and mRNA (Fig. 5B and C) was also pronounced in mice treated with scopolin. Similarly, Ajmo *et al*.^41^ observed increased hepatic SIRT activity, as well as protein and mRNA expression, in mice exhibiting improved phenotypes against alcoholic liver steatosis which was provoked by resveratrol, a potent natural agonist of SIRT1. A separate study demonstrated that adiponectin signaling increases SIRT1 protein expression levels in primary human myotubes^42^. Taken together, it is likely that the upregulation of hepatic SIRT1 activity and protein expression induced by scopolin administration in mice could be mediated partially through increased plasma adiponectin concentrations. Interestingly, mice administered scopolin, in the present study, exhibited markedly increased levels of circulating adiponectin (Fig. 3D), as well as hepatic AdipoR2 expression (Fig. 5C), compared with HFD-fed mice. The precise mechanism by which scopolin induces circulating adiponectin levels in HFD-fed mice remains to be elucidated. Lastly, we also cannot rule out the possibility that increased circulating adiponectin levels in mice fed scopolin may also influence hepatic lipid metabolism when adiponectin binds to its receptor, AdipoR2, eventually activating the AdipoR/LKB1/AMPK signaling cascade.

In summary, the present study suggests that scopolin, a coumarin compound isolated from *A. iwayomogi*, effectively reduces hepatic lipid accumulation through the activation of SIRT1-mediated signaling cascades in HFD-fed mice. Increased hepatic SIRT1 activity and protein levels appear to be associated with these beneficial effects of scopolin. Conducting further studies on the efficacy of scopolin in humans and on its pharmacokinetic profiles, it may serve as a novel and promising therapeutic agent in treating human non-alcoholic fatty liver.

## Methods

### Isolation of scopolin

The powder of *A. iwayomogi* (5 kg) was extracted using 50% (v/v) ethanol to obtain a dried ethanol extract of 0.7 kg (yield: 14%). This dried extract was suspended in water (1.5 L) and partitioned with chloroform (3 × 1.5 L) to obtain water-soluble (456.7 g) and water-insoluble (97.6 g) subfractions. The water-soluble fraction was further adsorbed onto a non-ionic porous resin (Amberlite XAD-2, Sigma-Aldrich, St. Louis, MO, USA) and desorbed with 30%, 60%, and 100% methanol. The 30% methanol fraction (92.2 g) was chromatographed on a Sephadex LH-20 gel (Sigma-Aldrich) column by using 40% methanol to yield five fractions (Fr 1–Fr 5). Fr 2 (24.1 g) was subjected to separation on an MCI CHP20P gel (Sigma-Aldrich) column by using 5% methanol to yield six fractions (Fr 1–Fr 6). Fr 2 was concentrated, yielding scopolin (4.15 g).

To identify the scopolin peak from the high-performance liquid chromatography (HPLC) chromatogram of *A. iwayomogi* extract, scopolin compound (0.1 mg) and dried 50% ethanol extract of *A. iwayomogi* (1 mg) were immersed in 1 mL aqueous 50% ethanol, and then filtered through a 0.2 µm membrane filter. HPLC analyses were performed using a Waters HPLC system (Waters Corporation, Milford, MA, USA) with an auto sampler. Chromatographic separation was achieved using a Sunfire C_18_ column (4.6 mm × 250 mm, 5 μm inner diameter, Waters) at 30 °C with a flow rate of 0.9 mL/min using a gradient mobile phase composed of 0.1% (v/v) aqueous formic acid (A) and acetonitrile (B) with a gradient elution condition of 85–84% A at 0–5 min, 84–72% A at 5–10 min, 72–69% A at 10–15 min, and 69–40% A at 15–20 min. The injection volume was 10 μL, and the detection wavelength was set at 330 nm.

### Cell culture and Oil-Red O staining

HepG2 human hepatic carcinoma cells (American Type Culture Collection, Manassas, VA, USA) were cultured at 37 °C in a 5% CO_2_-humidified incubator and grown in minimum essential medium supplemented with 10% fetal bovine serum, penicillin (100 U/mL) and streptomycin (100 μg/mL). Scopolin was dissolved in dimethyl sulfoxide (DMSO) at concentrations of 100 mM, and then added to the reaction mixture for a final concentration of 100 μM. To deposit lipids in HepG2 cells, the cells were incubated for 24 h with serum-free medium containing 1 mM oleic acid with fatty acid-free bovine serum albumin. Lipid accumulation in HepG2 cells was evaluated by Oil-Red O staining in the presence of the SIRT1 inhibitor EX527 (Sigma-Aldrich). Briefly, the cells were washed twice with phosphate-buffered saline (PBS, pH 7.4), and then fixed with 2 mL 10% formalin solution in PBS for 1 h at room temperature. After two rinses with PBS, the cells were stained with 0.5% Oil-Red O (Sigma-Aldrich) in isopropanol for 30 min with gentle agitation. Cells were washed twice with distilled water and photographed under a light microscope. Lipids of the cells were extracted in 2 mL 100% isopropanol and their optical density values were measured in a spectrophotometer at 600 nm.

### SIRT1 activity assay

SIRT1 activity was measured by using a SIRT1 Fluorometric Drug Discovery Kit (BML-AK555, Enzo Life Sciences International, Inc., Plymouth Meeting, PA, USA). In this assay, SIRT1 activity is assessed by the degree of deacetylation of a standardized substrate containing an acetylated lysine side chain. The substrate utilized is a peptide containing amino acids 379–382 of human p53 (Arg-His-Lys-Lys [Ac]), an established target of SIRT1 activity; SIRT1 activity is directly proportional to the degree of deacetylation of Lys-382. For SIRT1 activator screening, scopolin (100 µM) was added to a human recombinant SIRT1 enzyme and incubated with the peptide substrate (25 µM) and NAD^+^ (500 µM) in PBS at 37 °C on a horizontal shaker for 45 min. The reaction was stopped by adding 2 mM nicotinamide and a developing solution that binds to deacetylated lysine to form a fluorophore. Following a 10-min incubation at 37 °C, fluorescence was read in a fluorescence microplate reader (excitation, 360 nm; emission, 450 nm). For endogenous SIRT1 activity measurement, the assay was modified using nuclear extracts containing approximately 10 µg protein. The nuclear extracts from liver tissues or HepG2 cells were prepared by using the Nuclear Extract kit (Active Motif, Carlsbad, CA, USA) according to the manufacturer’s instructions. Data for endogenous SIRT1 activation were normalized to protein concentrations in the nuclear extracts measured using Bradford assay reagents (Bio-Rad, Hercules, CA, USA).

### Animal model and experimental protocol

Five-week-old male C57BL/6 N mice weighing 18–20 g were obtained from Orient Bio (Gyeonggi-do, Korea). Mice were housed in a pathogen-free facility at Yonsei University (Seoul, Korea) at room temperature with a 12-h light/dark cycle. All animal experiments were performed in accordance with the Korea Food and Drug Administration guidelines. All experimental protocols were reviewed and approved by the Institutional Animal Care and Use Committee of the Yonsei Laboratory Animal Research Center. During one week of acclimatization, animals were fed a commercial diet (Purina Rodent Chow 5001, Nestlé Purina, St. Louis, MO, USA) and tap water *ad libitum*. Thereafter, the mice were divided into three groups (n = 8/group) and fed one of three experimental diets: Normal diet (ND), HFD, and HFD supplemented with scopolin (SPD). The ND was a purified diet based on the AIN-76 rodent diet. The HFD was identical to the ND, except for the addition of 200 g fat/kg (170 g lard and 30 g corn oil) and 0.1% (w/w) cholesterol. The SPD was identical to the HFD, but also contained 0.02% (w/w) scopolin. All animals had access to diet and water *ad libitum*. Mice were kept on the treatment for 8 weeks. Weekly body weight and daily food intake were measured throughout the experimental period. At the end of the experiment, the mice were anaesthetized with 0.15 mL avertin (2.5% in tert-amyl alcohol) per 10 g body weight after a 12-hr fast. Blood was drawn from the abdominal aorta into an EDTA-coated tube, and plasma was obtained by centrifuging the blood at 2,000 × g for 15 min at 4 °C. The livers were excised, rinsed with PBS, and weighed, and a portion of each liver was fixed in 10% formalin for further analysis. Plasma and liver samples were stored at −80 °C until analysis.

### Biochemical analysis

Liver tissues were homogenized, and total hepatic lipids were extracted as described by Folch *et al*.^43^ and re-dissolved in 2 mL ethanol. Hepatic TG, cholesterol, and fatty acid contents were determined by using commercial kits (Bio-Clinical System, Gyeonggi-do, Korea). Plasma activities of aspartate aminotransferase (AST) and alanine aminotransferase (ALT) were measured by using commercial kits (Bio-Clinical System). Plasma levels of adiponectin, monocyte chemoattractant protein-1 (MCP-1), tumor necrosis factor-alpha (TNFα), and IL-6 were determined by using ELISA kits (Millipore, Billerica, MA, USA).

### Histological examination

Liver tissue specimens fixed in 10% buffered formalin were embedded in paraffin, cut into 5-μm-thick slices, and stained with hematoxylin and eosin (H&E) for histological examination of fat droplets. Steatosis and inflammation were numerically scored according to semi-quantitative pathological standards. Briefly, the pathological degree of steatosis was scored as follows: No steatosis = 0; minimal steatosis = 1; slight steatosis = 2; moderate steatosis = 3; marked steatosis = 4; severe steatosis = 5. In addition, the degree of lobular inflammation was scored as follows: No inflammation foci = 0; 1–2 inflammation foci = 1; 3–4 inflammation foci = 2; >4 inflammation foci = 3.

### Semiquantitative reverse transcription polymerase chain reaction (RT-PCR) analysis

Total mRNA from the liver samples or HepG2 cells was isolated using TRIzol reagent (Invitrogen, Carlsbad, CA, USA). RT-PCR was performed using a Superscript II kit (Invitrogen). Forward and reverse primer sets for target and internal marker genes are listed in Supplementary Table 1. RT-PCR was performed as follows: Five-minute initial denaturation at 94 °C; 35–38 cycles of 30-sec denaturation at 94 °C; 30-sec annealing at 55 °C and 1-min extension at 72 °C; 10-min final extension at 72 °C. Next, 4 μL of each PCR reaction mixture was mixed with 1 μL 6X loading buffer and loaded onto a 2% agarose gel containing ethidium bromide. Glyceraldehyde-3-phosphate dehydrogenase (GAPDH) mRNA levels were used as an internal control.

### Western blot analysis

Liver tissues were lysed in Western lysis buffer consisting of 100 mM Tris-HCl (pH 7.4), 5 mM EDTA, 50 mM NaCl, 50 mM sodium pyrophosphate, 50 mM NaF, 100 mM orthovanadate, 1% Triton X-100, 1 mM phenylmethylsulfonyl fluoride, 2 μg/mL aprotinin, 1 μg/mL pepstatin A, and 1 μg/mL leupeptin. Samples were incubated on ice with frequent vortexing for 10 min and centrifuged for 20 min at 1,300 × g. The protein concentration of each supernatant was quantified using a protein assay reagent from the Bradford assay (Bio-Rad) in accordance with the manufacturer’s instructions.

Proteins were loaded onto an 8% SDS-PAGE and transferred to a nitrocellulose membrane (Amersham, Buckinghamshire, UK). After transfer, membranes were blocked with 5% bovine serum albumin (BSA) in Tris-buffered saline with 0.05% Tween 20 and probed with the specified primary antibodies (diluted 1:1,000) overnight at 4 °C. Primary antibodies were sourced as follows: SIRT1, β-actin, and NF-κB p65 were obtained from Santa Cruz Biotechnology (Santa Cruz, CA, USA); Ac-NF-κB p65, AMPK, phospho (p)-AMPK (Thr172), mTOR, p-mTOR (Thr2448), S6 Kinase 1 (S6K1), p-S6K1 (Thr389), ACC, and p-ACC (Ser79) were obtained from Cell Signaling Technology (Danvers, MA, USA). After washing, the membranes were incubated with secondary antibodies in Tris-buffered saline with 0.05% Tween 20 for 1 hr. The blots were then developed using an ECL detection kit (Amersham) according to the manufacturer’s instructions.

### Coimmunoprecipitation

Nuclear extracts from liver tissues were brought to a final volume of 1 mL with buffer containing 10 mM PBS, 50 mM KCl, 0.05 mM EDTA, 2.5 mM MgCl_2_, 8.5% glycerol, 1 mM dithiothreitol, 0.1% Triton X-100, 2% BSA, and 1 mg/mL nonfat milk for 6 hr at 4 °C, and incubated with 2 μg of SREBP1c, PGC-1α, or LKB1 antibody (Santa Cruz Biotechnology). The immunocomplex was captured by incubating the samples with protein A-agarose (Santa Cruz Biotechnology) suspension overnight at 4 °C on a rocking platform. Agarose beads were collected by centrifugation and washed three times with PBS containing protease inhibitors. After microcentrifugation, the pellet was washed with 30 μL SDS-PAGE sample buffer and boiled for 5 min at 100 °C. An aliquot of the supernatant was subjected to electrophoresis on 10% SDS-PAGE and immunoblotted with an antibody against acetyl-lysine (Cell Signaling Technology).

### Statistical analysis

All data are expressed as means ± SEM. Statistical analysis was performed using one-way ANOVA and analyzed further by Duncan’s multiple range test for statistical difference. Differences among experimental groups were considered to be statistically significant at *p* < 0.05.

## Electronic supplementary material

Supplementary Information
