# Supplementary material for: Scopolin ameliorates high-fat diet induced hepatic steatosis in mice: potential involvement of SIRT1-mediated signaling cascades in the liver
Source: Sci Rep. 2017 May 22;7:2251. doi: 10.1038/s41598-017-02416-6 (PMC5440403; doi:10.1038/s41598-017-02416-6)
Supplement: Supplementary file 1 — Supplementary Information [file 41598_2017_2416_MOESM1_ESM.pdf]

Supplementary Information

Scopolin ameliorates high-fat diet induced hepatic steatosis in mice: potential involvement of SIRT1-mediated signaling cascades in the liver

Ahyoung Yoo[1], Vikram P. Narayan[1], Eun Young Hong[2], Wan Kyunn Whang[2], and Taesun Park[1,*]

[1]Department of Food and Nutrition, Brain Korea 21 PLUS Project, Yonsei University, 50 Yonsei-ro, Seodaemun-gu, Seoul 03722, South Korea

[2]Pharmaceutical Botany Laboratory, College of Pharmacy, Chung-Ang University, Heukseok-dong, Dongjak-gu, Seoul 06974, South Korea

***Corresponding author**: Taesun Park, Department of Food and Nutrition, Yonsei University, 50 Yonsei-ro, Seodaemun-gu, Seoul 03722, South Korea

E-mail address: tspark@yonsei.ac.kr; Tel.: +82-2-2123-3123; Fax: +82-2-365-3118

Supplementary Table 1. Primer sequences used for semi-quantitative RT-PCR analysis

Gene description	Primers	Sequences (5'→3')	Annealing temperature (°C)	PCR product (bp)
Liver X receptor α (LXRα)	F<br>R	TCCTACACGAGGATCAAGCG<br>AGTCGCAATGCAAAGACCTG	55	119
Sterol regulatory element-binding factor 1c (SREBP1c)	F<br>R	ATCGCAAACAAGCTGACCTG<br>AGATCCAGGTTTGAGGTGGG	55	115
Acetyl-CoA carboxylases (ACC)	F<br>R	GCCATTGGTATTGGGGCTTAC<br>CCCGACCAAGGACTTTGTTG	55	119
Lipoprotein lipase (LPL)	F<br>R	ACTTGTCATCTCATTCCTGG<br>GCACCCAACTCTCATACATT	55	166
Fatty acid synthase (FAS)	F<br>R	AGGTATGGCCACTTTGGGAC<br>GCTGGTCTTGCTGTGCATCT	55	103
Adiponectin receptor 2 (AdipoR2)	F<br>R	ATGGTGGCGCTTTTCTCTGG<br>GCACAGGTCTTGCAAATGGC	55	136
Sirtuin 1 (SIRT1)	F<br>R	AGCTCCTTGGAGACTGCGAT<br>TGGGCGTGGAGGTTTTTCAG	55	122
Peroxisome proliferator-activated receptor alpha (PPARα)	F<br>R	TGGGGATGAAGAGGGCTGAG<br>ACGCAACGTAGAGTGCTGTG	55	197
Carnitine palmitoyltransferase 1 (CPT1)	F<br>R	GCCACCCAGAGGAGATTGAT<br>CCCAGGACTGGTTCATCCTT	55	110
Monocyte chemotactic protein-1 (MCP-1)	F<br>R	GGGCAAGCTAGGCTGTGTTT<br>TAGCCACTCCTGGGAGCAAA	55	197
Tumor necrosis factor-alpha (TNFα)	F<br>R	CCCTCAGCAAACCACCAAGT<br>ACAAGGTACAACCCATCGGC	55	128
Interleukin-6 (IL-6)	F<br>R	TCCAGTTGCCTTCTTGGGAC<br>ACAGGTCTGTTGGGAGTGGT	55	105
Glyceraldehyde-3-phosphate dehydrogenase (GAPDH)	F<br>R	CGTCTAGAAAAACCTGCCAA<br>ATGAGCTTGACAAAGTGGTC	55	191

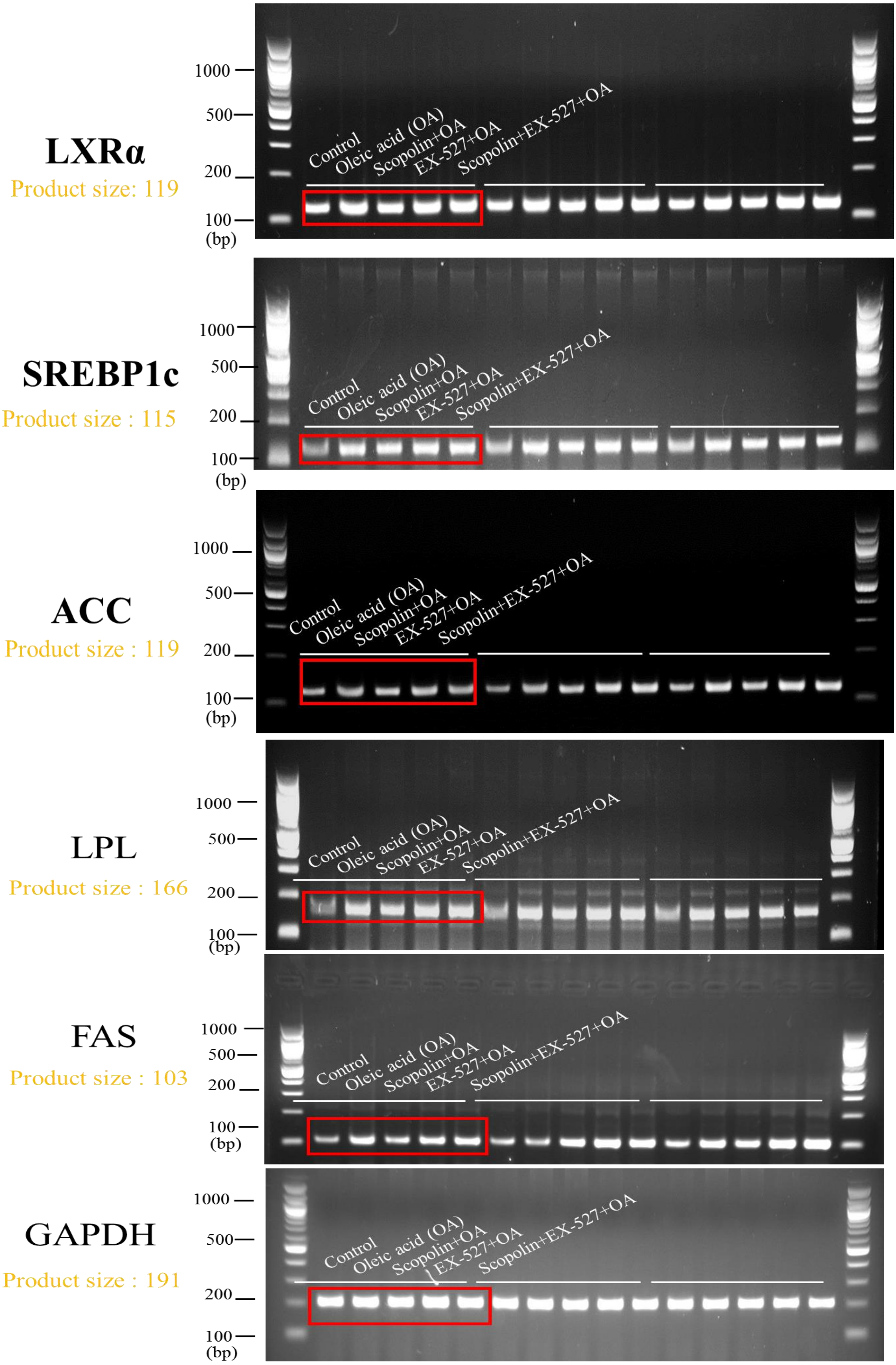
LXRα
Product size: 119
SREBP1c
Product size : 115
ACC
Product size : 119
LPL
Product size : 166
FAS
Product size : 103
GAPDH
Product size : 191
1000
500
200
100
(bp)
Control
Oleic acid (OA)
Scopolin+OA
EX-527+OA
Scopolin+EX-527+OA

Supplementary Figure S1. Full length agarose gels relative to Fig 2C. Red boxes indicate the cropping lines used to generate the figures.

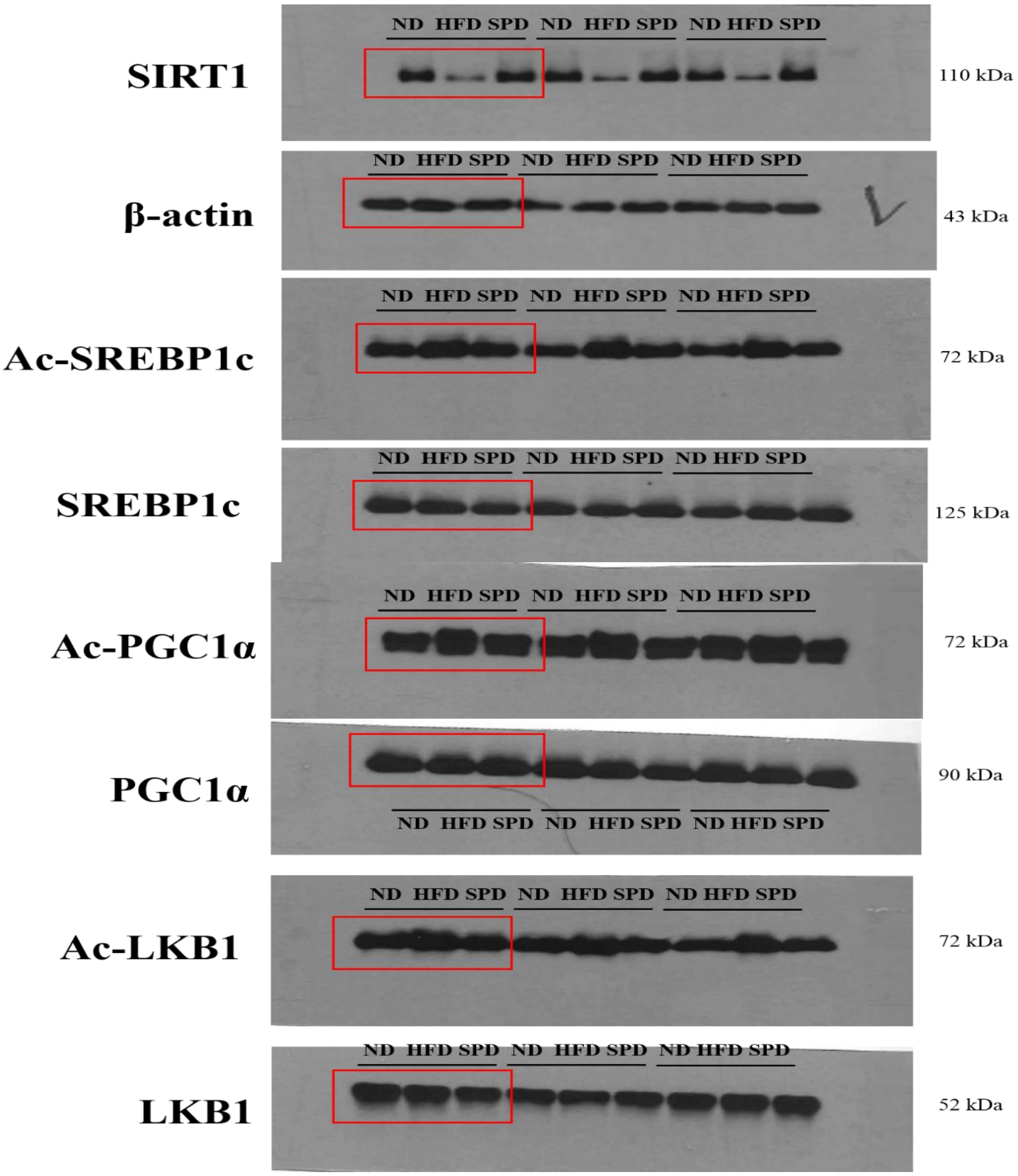

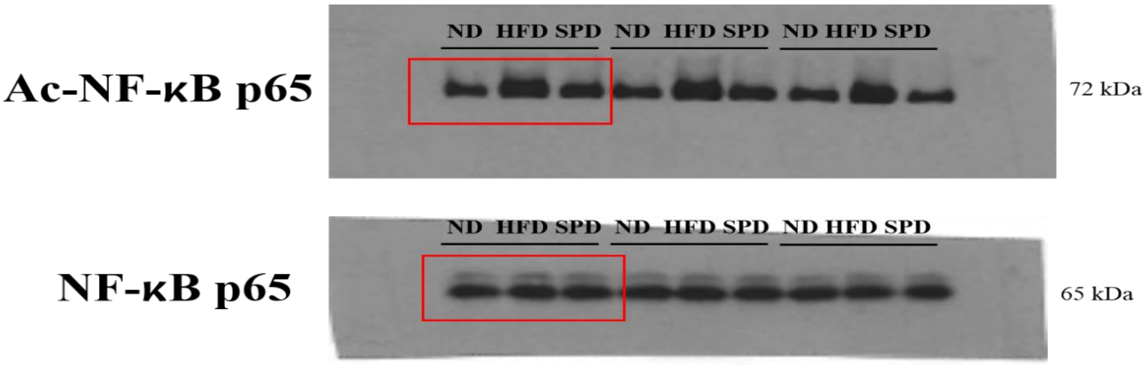

Supplementary Figure S2. Full length western blot membranes relative to Fig 5B. Red boxes indicate the cropping lines used to generate the figures.

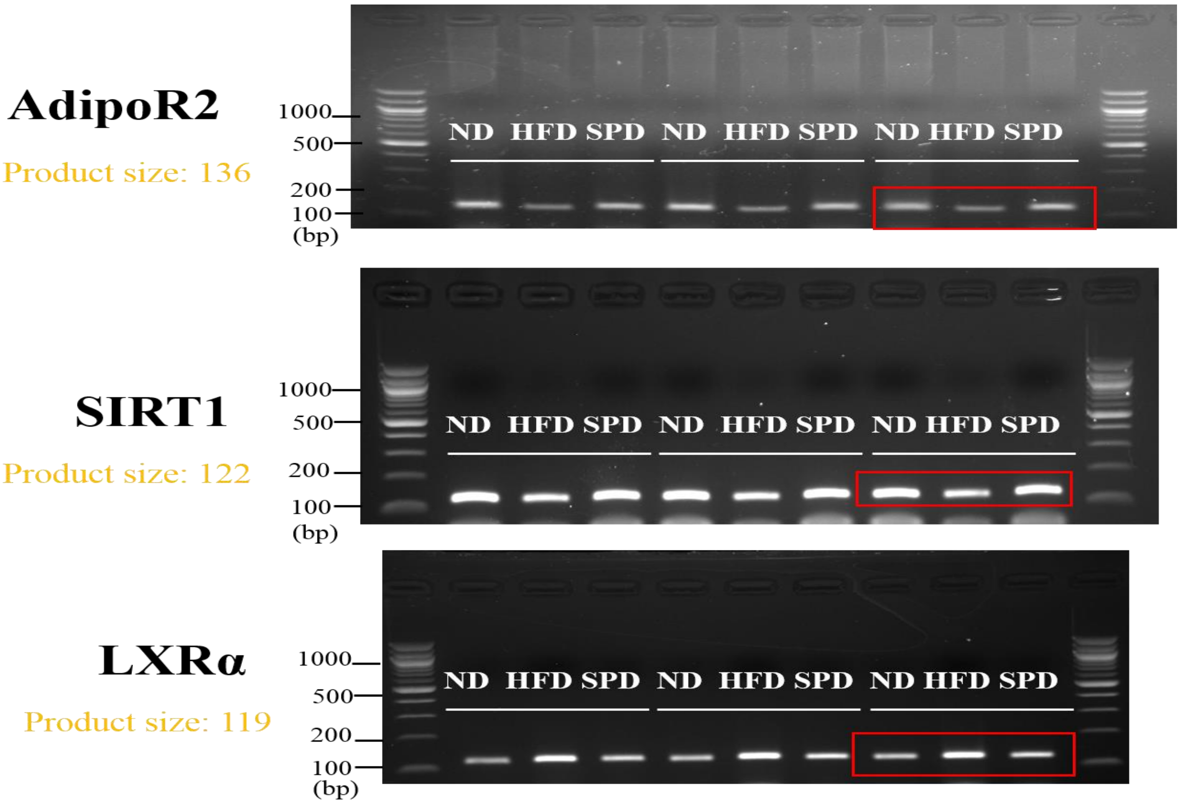

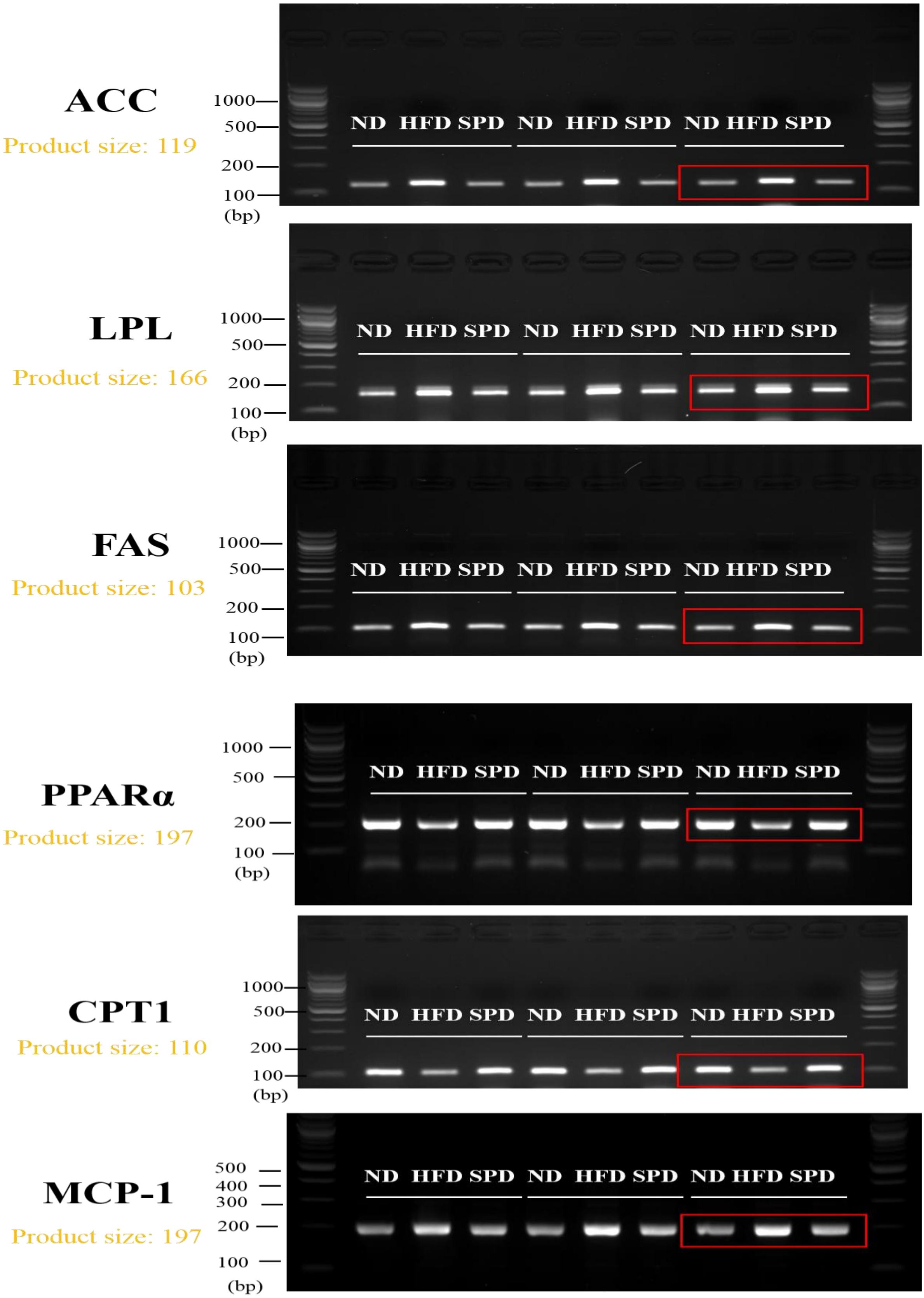

ACC
Product size: 119
1000
500
200
100
(bp)
ND HFD SPD ND HFD SPD ND HFD SPD
LPL
Product size: 166
1000
500
200
100
(bp)
ND HFD SPD ND HFD SPD ND HFD SPD
FAS
Product size: 103
1000
500
200
100
(bp)
ND HFD SPD ND HFD SPD ND HFD SPD
PPARα
Product size: 197
1000
500
200
100
(bp)
ND HFD SPD ND HFD SPD ND HFD SPD
CPT1
Product size: 110
1000
500
200
100
(bp)
ND HFD SPD ND HFD SPD ND HFD SPD
MCP-1
Product size: 197
500
400
300
200
100
(bp)
ND HFD SPD ND HFD SPD ND HFD SPD

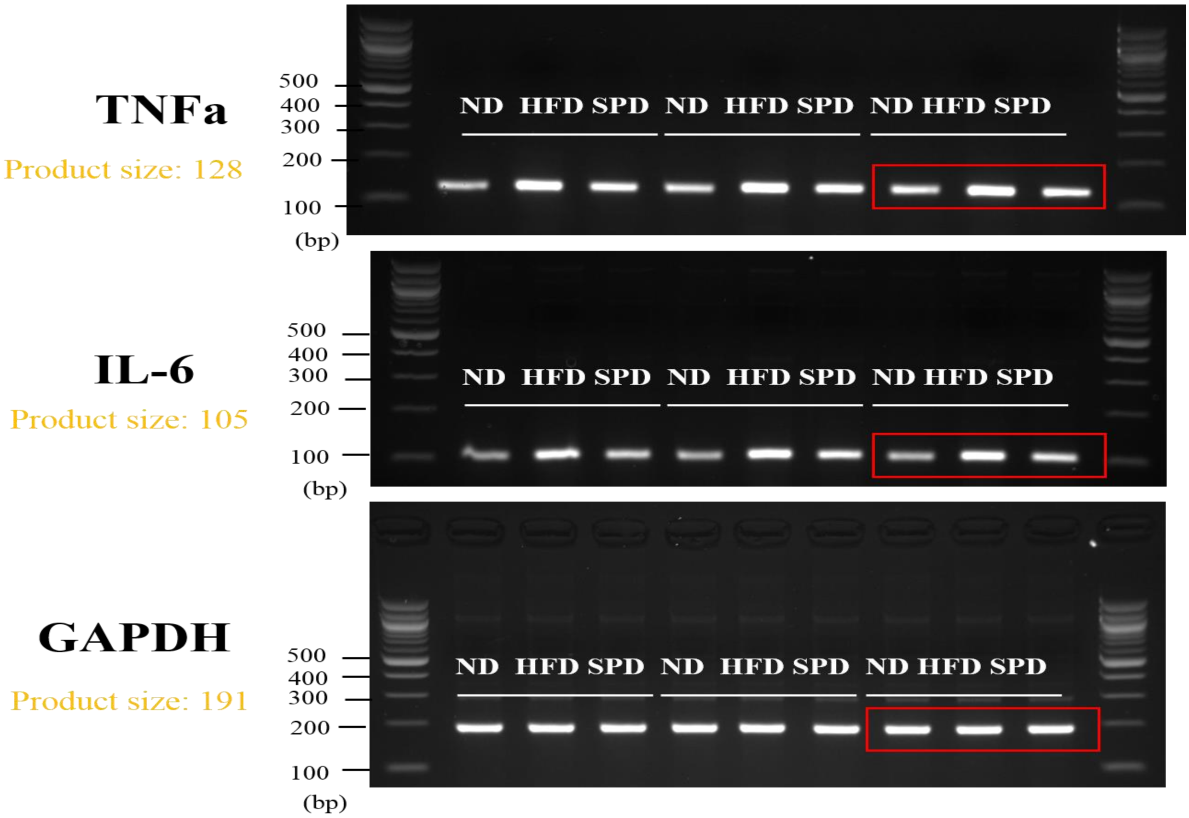

Supplementary Figure S3. Full length agarose gels relative to Fig 5C. Red boxes indicate the cropping lines used to generate the figures.

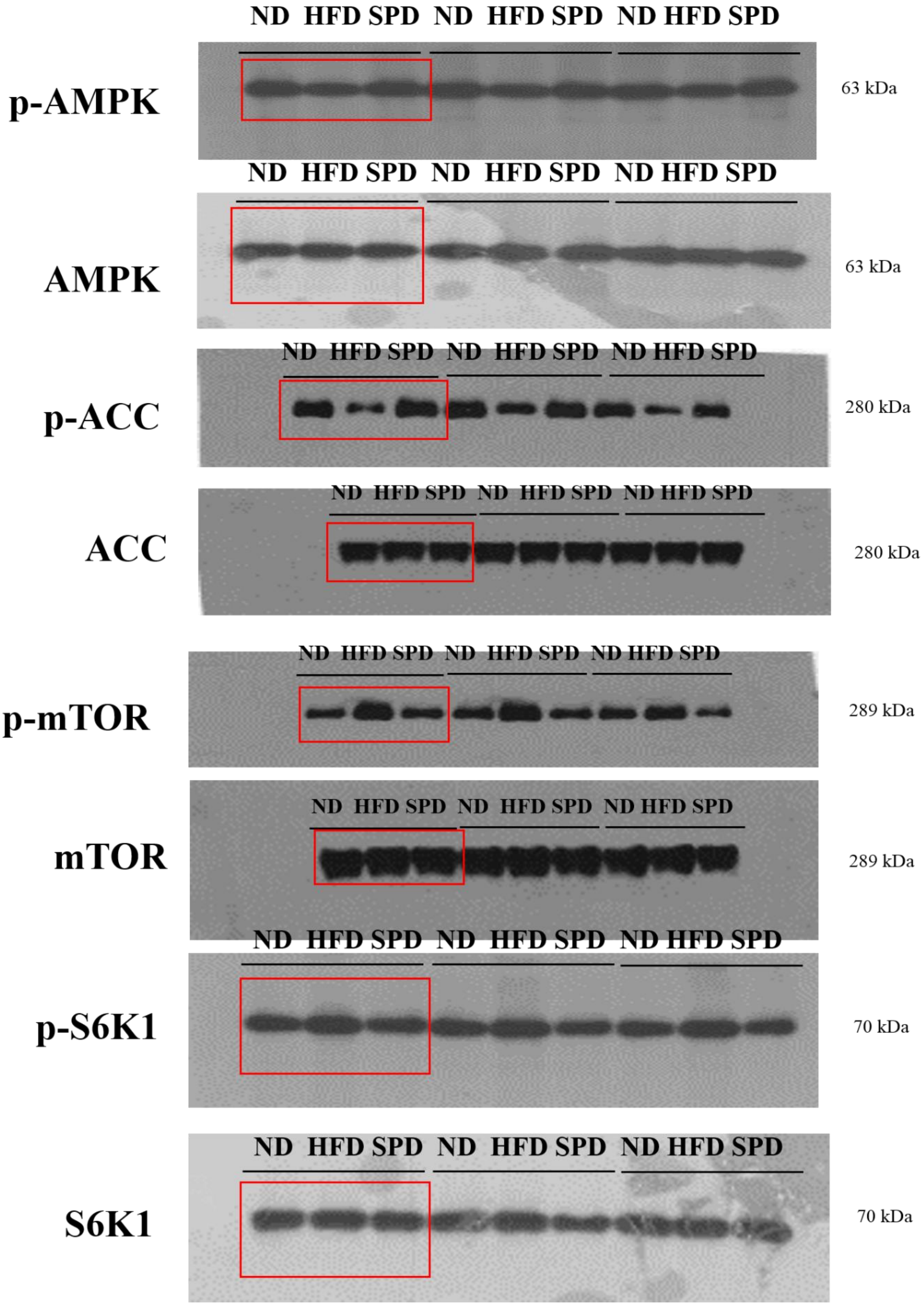

Supplementary Figure 4S. Full length western blot membranes relative to Fig 5D. Red boxes indicate the cropping lines used to generate the figures.
